# Prognostic value of histopathology and trends in cervical cancer: a SEER population study

**DOI:** 10.1186/1471-2407-7-164

**Published:** 2007-08-23

**Authors:** Vincent Vinh-Hung, Claire Bourgain, Georges Vlastos, Gábor Cserni, Mark De Ridder, Guy Storme, Anne-Thérèse Vlastos

**Affiliations:** 1 Oncologisch Centrum, Universitair Ziekenhuis Brussel, 101 Laarbeeklaan, 1090 Jette, Brussels, Belgium; 2 Geneva Cancer Registry, Institute for Social and Preventive Medicine, University of Geneva, Geneva, Switzerland; 3 Hôpitaux Universitaires de Genève, Geneva, Switzerland; 4 Cserni BT and Bács-Kiskun County Teaching Hospital, Kecskemét, Hungary

## Abstract

**Background:**

Histopathology is a cornerstone in the diagnosis of cervical cancer but the prognostic value is controversial.

**Methods:**

Women under active follow-up for histologically confirmed primary invasive cervical cancer were selected from the United States Surveillance, Epidemiology, and End Results (SEER) 9-registries public use data 1973–2002. Only histologies with at least 100 cases were retained. Registry area, age, marital status, race, year of diagnosis, tumor histology, grade, stage, tumor size, number of positive nodes, number of examined nodes, odds of nodal involvement, extent of surgery, and radiotherapy were evaluated in Cox models by stepwise selection using the Akaike Information Criteria.

**Results:**

There were 30,989 records evaluable. From 1973 to 2002, number of cases dropped from 1,100 new cases/year to 900/year, but adenocarcinomas and adenosquamous carcinoma increased from 100/year to 235/year. Median age was 48 years. Statistically significant variables for both overall and cause-specific mortality were: age, year of diagnosis, race, stage, histology, grade, hysterectomy, radiotherapy, tumor size and nodal ratio. The histological types were jointly significant, P < 0.001. Cause-specific mortality hazard ratios by histological type relatively to non-microinvasive squamous cell carcinoma were: microinvasive squamous cell carcinoma 0.28 (95% confidence interval: 0.20–0.39), carcinoma not otherwise specified 0.91 (0.79–1.04), non-mucinous adenocarcinoma 1.06 (0.98–1.15), adenosquamous carcinoma 1.35 (1.20–1.51), mucinous adenocarcinoma 1.52 (1.23–1.88), small cell carcinoma 1.94 (1.58–2.39).

**Conclusion:**

Small cell carcinoma and adenocarcinomas were associated with poorer survival. The incidental observation of increasing numbers of adenocarcinomas despite a general decline suggests the inefficiency of conventional screening for these tumors. Increased incidence of adenocarcinomas, their adverse prognosis, and the young age at diagnosis indicate the need to identify women who are at risk.

## Background

Cancer of the cervix uteri is the second most common cancer (after breast cancer) and the third leading cancer mortality (after lung and breast cancer) among women worldwide [1]. Squamous cell carcinoma (SCC) is the predominant histological type accounting for three-fourths of all cervical cancers. Adenocarcinoma and adenosquamous cell carcinoma represent 10–15%, and other or unspecified histology represent the remaining 10–15% [2,3]. There is controversy regarding whether or not these different histological types have any bearing on prognosis [4-7]. Some authors found that adenocarcinoma had a poor prognosis [8,9], others found no evidence of pathological type as a risk factor [6,10]. Since histopathology is a cornerstone in the detection and the diagnosis of cervical cancer, we believe that clarifying the prognostic value of pathological type is an important issue that might influence the management, treatment and surveillance planning of newly diagnosed cervical cancer. The present study uses the United States Surveillance, Epidemiology, and End Results (SEER) population data in order to evaluate the prognostic role of histopathological type in invasive carcinoma of the cervix.

## Methods

Records of patients under active follow-up were abstracted from the 9-registries of the SEER [11]. Selected patients were women with histologically confirmed primary invasive cervical cancer diagnosed between 1973 and 2002. Tumor histopathologies were classified according to the SEER's implementation of the International Classification of Diseases for Oncology (ICDO), Second Edition [11]. Only histopathological types with at least 100 cases were retained.

Univariate analyses of overall survival (event = death from any cause) and cause-specific survival (event = death from cervical cancer) used the Kaplan-Meier method [12].

Multivariate analyses used the proportional hazards models. The selection of variables combined backward and forward stepwise selection by minimizing the Akaike Information Criteria (AIC) [13]. The AIC is computed as minus twice the log likelihood plus twice the number of parameters of the model. The AIC penalizes over-parameterization, variables are retained only when the model improves enough to balance the number of parameters. The variables evaluated were: SEER registry area, age, marital status, race, tumor histopathology, grade, historical stage (localized versus regional, metastatic, or unknown), extent of surgery (hysterectomy versus else), radiotherapy (prescribed versus none or unknown). Up to four levels of interactions were considered in the stepwise selection. More detailed pathological data and AJCC (American Joint Committee on Cancer) stage was available in a subset of patients. A second analysis was done on that subset with the detailed variables: AJCC stage, tumor size, number of removed nodes, number of positive nodes, and log odds of nodal involvement computed as Log ((number of positive nodes + 0.5)/(number of negative nodes + 0.5)) [14]. The log odds was used to generalize the lymph node ratio to node-negative cases. The assumption of proportional hazards were checked using the Schoenfeld residuals method [15] [see Additional File 1]. The linearity of the functional forms of continuous variables were verified using the martingale residuals [15] [see Additional File 2].

For purpose of condensing the report, histopathological types were grouped as: carcinoma not otherwise specified (ICDO 8010), squamous cell carcinoma microinvasive (8076), non-microinvasive squamous cell carcinoma (8070, 8071, 8072), adenosquamous carcinoma (8560), adenocarcinoma excluding mucinous (8140, 8260, 8310, 8380), mucinous adenocarcinoma (8480, 8481), and small cell carcinoma (8041).

## Results

### 1. Trends and characteristics

**Figure 1:**
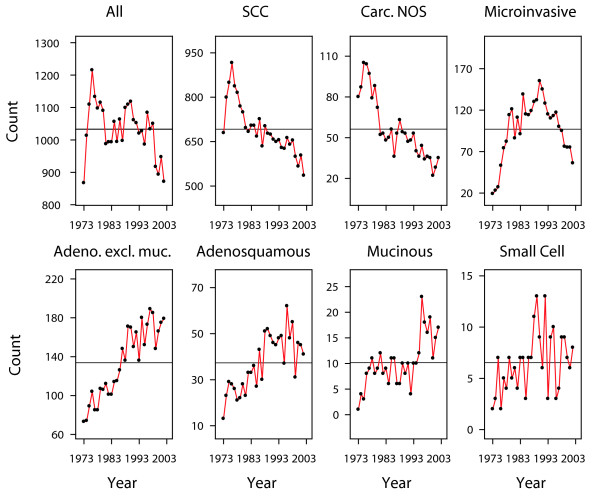
**Numbers of new cervical cancers, by histopathological type**. All graphs are centered around the mean number of cases per year (horizontal line). The vertical axes are scaled to highlight the trend within each histopathological type.

There were 32,040 cases of histologically confirmed primary invasive cancer of the cervix and 81 histological types. Among these, 30,989 records matched the selection criteria, representing the study population, with 13 histological types. The median follow-up was 10.5 years. The predominant histopathological types were invasive squamous cell carcinomas (SCC) accounting for two thirds of the incidence (n = 20,755). There were notable changes in the counts of recorded cases over the last three decades. The overall number of cases diagnosed each year declined from an average of 1,100 cases per year (peak 1,109–1,215 per year in 1975–1976) to an average of 900 cases per year (893–871 in 2000–2002) (Figure 1). In terms of crude incidence rates, these correspond to a decline from 11.1 to 6.6 cases per 100,000 women per year. Most of the decline could be attributed to the decline in the incidence of SCC (Figure 1). The yearly count of carcinoma not otherwise specified (NOS) also declined from an average of 95 per year in 1975 to an average of 30 per year in 2000. However, the yearly count of adenocarcinomas increased: non-mucinous from an average of 80 per year (73–89 in 1973–1975) to 175 per year (166–179 in 2000–2002), adenosquamous from an average of 20 per year (13–29 in 1973–1975) to 45 per year (46–41 in 2000–2002), mucinous from almost none (1–3 cases per year in 1973–1975) to 15 per year (11–17 in 2000–2002) (Figure 1).

Table 1 shows the distribution of patients and tumor characteristics according to the histopathology group. Women were young in all histological groups. The lowest median age was 38 years in microinvasive SCC, the highest median age was 50 years in SCC and in mucinous adenocarcinoma, the overall median age was 48 years. By race, the distribution of histological types were comparable between White Non-Hispanic and White Hispanic women. The distribution differed in African-American women in whom squamous cell types were relatively more frequent than adenocarcinomas. By tumor characteristics, non-mucinous adenocarcinomas were associated with smaller tumor size (median 25 mm), less nodal involvement (log odds -3.6), less advanced stage (71% stage I, 10% stage II) as compared with SCC (median tumor size 40 mm, log odds nodal involvement -3.4, 51% stage I, 19% stage II). Small cell carcinoma was associated with the most advanced tumor involvement (median tumor size 45 mm, log odds nodal involvement -2.7, 32% stage I, 14% stage II). Adenosquamous and mucinous carcinomas were associated with intermediary characteristics. Information on surgery was available in 17,994 records. Hysterectomy was generally performed in more than half of the cases. Information on whether or not radiotherapy had been prescribed was available in 30,747 records. Radiotherapy was performed in about 60% of these cases, except microinvasive SCC and unspecified carcinoma for which respectively only 4% and 26% received some form of radiotherapy.

**Table 1:** Invasive tumors: patient and tumor characteristics by histological type

**Characteristic**	**Non-missing data**	**Microinvasive SCC**	**Carcinoma not otherwise specified**	**Squamous Cell (SCC)**	**Adenoca. excl. mucinous**	**Adeno-squamous**	**Mucinous adeno-carcinoma**	**Small cell carcinoma**
		n = 2910	n = 1688	n = 20755	n = 4015	n = 1120	n = 305	n = 196
Age ^(1)^	30989	38 (31~48)	42 (32~58)	50 (39~64)	47 (37~62)	44 (36~57)	50 (41~66)	48 (35~63)
Marital status (married)^(2)^	27999	1451 (55%)	736 (52%)	9193 (49%)	2102 (58%)	548 (53%)	160 (57%)	95 (51%)
Race	30764							
White non-hisp ^(2)^		2041 (71%)	1191 (72%)	13797 (67%)	3066 (77%)	785 (70%)	229 (75%)	127 (65%)
White hispanic ^(2)^		199 (7%)	96 (6%)	1671 (8%)	236 (6%)	75 (7%)	19 (6%)	14 (7%)
Black ^(2)^		362 (13%)	278 (17%)	3430 (17%)	318 (8%)	157 (14%)	23 (8%)	21 (11%)
Other ^(2)^		279 (10%)	95 (6%)	1735 (8%)	357 (9%)	97 (9%)	34 (11%)	32 (16%)
Tumor size (mm) ^(1)(3)^	6352	1 (1~2)	2 (1~53)	40 (17~60)	25 (10~45)	30 (20~50)	29 (20~47)	45 (29~69)
High histological grade ^(2)^	16378	95 (28%)	248 (86%)	5722 (49%)	821 (28%)	485 (70%)	61 (26%)	142 (100%)
Number of nodes removed ^(1)(4)^	14178	0 (0-0)	0 (0-0)	0 (0–9)	0 (0–17)	1 (0–18)	5 (0–21)	0 (0–11)
Node-positive status ^(2)^	5243	1 (0%)	9 (18%)	679 (21%)	143 (12%)	92 (24%)	33 (29%)	21 (48%)
Log odds nodal involvement ^(1)(5)^	4497	-3.4 (-3.9~-2.9)	-3.1 (-3.7~-1.9)	-3.4 (-3.9~-2.5)	-3.6 (-3.9~-2.9)	-3.3 (-3.8~-2.3)	-3.4 (-3.9~-1.9)	-2.7 (-3.7~-1.2)
AJCC Stage ^(6)^								
I ^(2)^	8831	1583 (99%)	373 (70%)	4674 (51%)	1657 (71%)	407 (60%)	100 (54%)	37 (32%)
II ^(2)^	2104	11 (1%)	51 (10%)	1708 (19%)	226 (10%)	74 (11%)	18 (10%)	16 (14%)
III ^(2)^	2223	7 (0%)	50 (9%)	1771 (20%)	216 (9%)	115 (17%)	35 (19%)	29 (25%)
IV ^(2)^	1358	4 (0%)	61 (11%)	925 (10%)	226 (10%)	77 (11%)	31 (17%)	34 (29%)
Hysterectomy ^(2)^	17994	1553 (72%)	364 (46%)	5302 (48%)	1966 (68%)	540 (67%)	146 (69%)	63 (47%)
Radiotherapy ^(2)^	30747	128 (4%)	435 (26%)	13084 (64%)	1819 (46%)	643 (58%)	183 (60%)	120 (62%)

^(1) ^Values are: median (lower ~ upper quartiles).

^(2) ^Values are: number of patients (%). Rounded percentages are computed excluding cases with missing data.

^(3) ^Tumor size: 1 = microscopic focus or foci; 2 = non-microscopic but <= 2 mm.

^(4) ^9678 cases 0 node examined, 4500 cases with >= 1 node examined.

^(5) ^Log ((number of positive nodes +0.5)/(number of negative nodes + 0.5)). More negative values indicate lower risk of nodal involvement [14].

^(6) ^SEER modified stage grouping: Nx cases (unknown nodal status) are grouped according to the T-stage.

### 2. Unadjusted survival analyses

**Figure 2:**
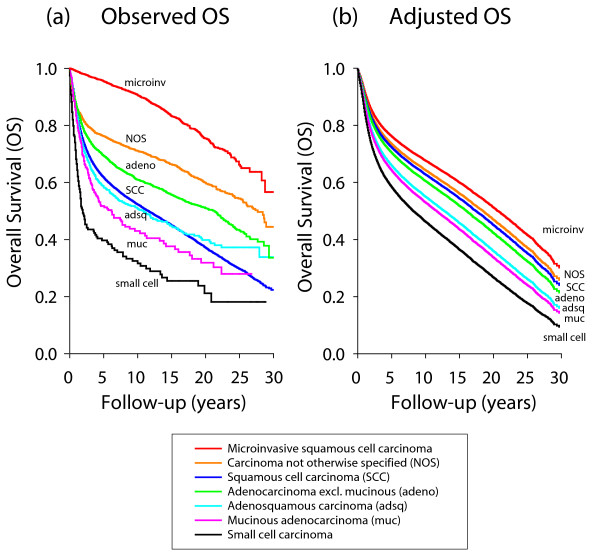
**Overall survival by histopathology group**. (a) Unadjusted Kaplan-Meier survival estimates. (b) Expected survival after adjustment taking into account registry area, age, year of diagnosis, race, marital status, grade, stage, surgery and radiotherapy, for a theoretical patient presenting with average characteristics of the population.

The 10-year overall survival was 57.8% (95% confidence interval 57.2%–58.4%) and the 10-year cause-specific survival was 74.2% (73.7%–74.8%). There were large statistically significant differences of overall survival between the histological groups (P < 0.0001) (Figure 2-a). The longest overall survival was observed with microinvasive SCC (90.6% at 10-year) and the poorest survival was observed with small cell carcinoma (31.6% at 10-year) (Table 2 and Figure 2-a). Between these two groups, the ranking from longer to shorter survival was: carcinoma NOS, non-mucinous adenocarcinoma, invasive SCC, adenosquamous carcinoma, and mucinous adenocarcinoma. The ranking of the histopathological groups by cause-specific survival was comparable, from the best cause-specific survival observed with microinvasive SCC (98.7% at 10-year), to the poorest survival observed with small cell carcinoma (44.5%) (Table 2).

**Table 2:** Kaplan-Meier estimates of overall survival and cause-specific survival by histopathology

**Histology**	**ICDO 4-digits**	**n**	**10 year overall survival (95% confidence interval)**	**10 year cause-specific survival (95% confidence interval)**
***All***		30989	57.8 (57.2–58.4)	74.2 (73.7–74.8)

***Carcinoma not otherwise specified (NOS)***	8010	1688	71.1 (68.9–73.4)	84.9 (83.1–86.7)
***Squamous cell carcinoma microinvasive***	8076	2910	90.6 (89.4–91.8)	98.7 (98.2–99.1)
***Squamous cell carcinoma (SCC)***		20755	52.3 (51.5–53.0)	69.7 (69.1–70.4)
SCC nonkeratinizing, NOS	8072	2692	54.9 (52.9–57.0)	69.1 (67.1–71.0)
SCC large cell, keratinizing	8071	2373	54.4 (52.2–56.6)	69.0 (66.9–71.1)
SCC NOS	8070	15690	51.6 (50.7–52.4)	70.0 (69.2–70.8)
***Adenosquamous carcinoma***	8560	1120	50.7 (47.5–53.9)	66.6 (63.5–69.7)
***Adenocarcinoma excluding mucinous***		4015	60.9 (59.2–62.6)	77.9 (76.4–79.3)
Endometrioid carcinoma	8380	198	68.9 (59.9–78.0)	87.9 (80.7–95.1)
Adenocarcinoma NOS	8140	3302	61.5 (59.7–63.4)	77.3 (75.7–79.0)
Papillary adenocarcinoma, NOS	8260	348	58.9 (53.4–64.4)	81.9 (77.3–86.4)
Clear cell adenocarcinoma, NOS	8310	167	47.1 (38.6–55.5)	68.2 (59.9–76.4)
***Mucinous adenocarcinoma***		305	43.0 (36.5–49.5)	62.1 (55.4–68.8)
Mucous adenocarcinoma	8480	181	43.6 (34.2–53.1)	64.1 (54.7–73.6)
Mucin-secreting adenocarcinoma	8481	124	41.3 (32.1–50.5)	59.1 (49.2–68.9)
***Small cell carcinoma NOS***	8041	196	31.6 (24.5–38.8)	44.5 (36.7–52.4)

### 3. Multivariate analysis

In a multivariate analysis for overall mortality, the variables retained by the stepwise selection were: SEER registry area, age at diagnosis, year of diagnosis, race, marital status, histological type, histological grade, stage, type of surgery, radiotherapy, and interactions between stage, surgery, and radiotherapy [see Additional File 3]. Figure 2-b shows the survival according to the histopathological group that would have been expected after adjustment by that stepwise selection. For cause-specific mortality, the SEER registry area and marital status were not significant while all other variables were significant (Table 3). Histology was among the most significant factors, regardless of whether the analysis was done using grouped histologies or whether the analysis was done using the distinct histological subtypes.

**Table 3:** Multivariate analysis of cause-specific mortality based on 30,989 cases of invasive cancer of the cervix

**Variable**	**Hazard ratio^(3) ^(95% confidence interval)**	**P**
**Demographic characteristics**		
SEER area		
Central registries	0.95 (0.89–1.01)	0.099
Western registries	0.97 (0.91–1.03)	0.308
Age at diagnosis	1.01 (1.00–1.01)	<0.001
Year of diagnosis	1.00 (1.00–1.01)	0.013
African-American ethnicity ^(1)^	1.14 (1.07–1.22)	<0.001
Marital status (married) ^(1)^	0.99 (0.94–1.04)	0.638

**Pathology**		
Histological type ^(2)^		<0.001
SCC microinvasive	0.28 (0.20–0.39)	<0.001
Carcinoma not otherwise specified	0.91 (0.79–1.04)	0.168
Adenocarcinoma excl. mucinous	1.06 (0.98–1.15)	0.126
Adenosquamous carcinoma	1.35 (1.20–1.51)	<0.001
Mucinous	1.52 (1.23–1.88)	<0.001
Small cell	1.94 (1.58–2.39)	<0.001
Histological High grade ^(1)^	2.17 (1.93–2.44)	<0.001
Localized stage ^(1)^	0.07 (0.05–0.08)	<0.001

**Treatments and interactions**		
Hysterectomy ^(1)^	0.26 (0.21–0.31)	<0.001
Radiotherapy ^(1)^	1.01 (0.92–1.11)	0.810
Hysterectomy * Radiotherapy	2.05 (1.67–2.52)	<0.001
High grade * Radiotherapy	0.60 (0.53–0.69)	<0.001
Localized stage * Hysterectomy	2.87 (2.17–3.78)	<0.001
Localized stage * Radiotherapy	5.24 (4.36–6.29)	<0.001
Localized stage * Hysterectomy * Radiotherapy	0.40 (0.28–0.56)	<0.001

^(1) ^Binarized variable, coded 1, versus all other levels of the variable including missing.

^(2) ^Reference level = non-microinvasive squamous cell carcinoma (SCC), all types.

^(3) ^Hazard ratio >1 indicates increased risk of dying from cervical cancer.

**Figure 3:**
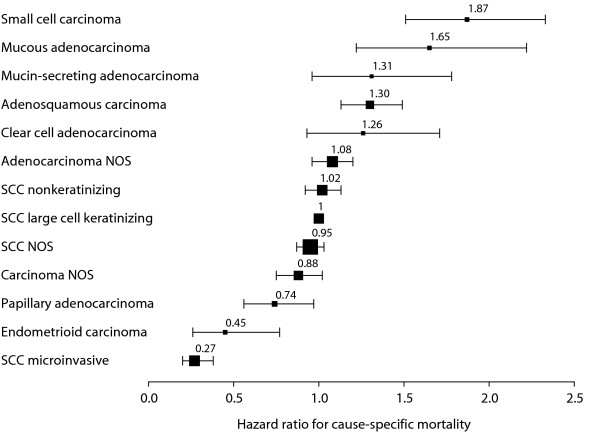
**Ranking of histopathological types by cause-specific mortality hazard ratios**. The hazard ratios were computed taking into account registry area, age, year of diagnosis, race, marital status, grade, stage, surgery, and radiotherapy. Horizontal bars: 95% confidence interval. Size of the boxes drawn as a function of the number of patients.

The ranking of the histological groups from best to poorest overall survival were: SCC microinvasive (hazard ratio HR = 0.28), carcinoma NOS (HR = 0.91), SCC non-microinvasive (reference, HR = 1), non-mucinous adenocarcinoma (HR = 1.06), adenosquamous (HR = 1.35), mucinous (HR = 1.52), and small cell carcinoma (HR = 1.94). The results of the same analysis but using the distinct histological types instead of groups are shown graphically in Figure 3. The hazard ratios ranged from the lowest risk histological types (SCC microinvasive, endometrioid, and papillary adenocarcinoma), through the intermediary risk types (the three SCC's and the adenocarcinoma NOS), to the highest risk types (clear cell adenocarcinoma, adenosquamous, mucin-secreting, mucous adenocarcinoma, and small cell carcinoma) (Figure 3).

### 4. Analyses on reduced subsets

There were 2,458 cases with information on tumor size, nodal involvement, and with non-missing AJCC staging. Table 4-A shows the results of the cause-specific analysis by stepwise selection applied to this subset of patients with more complete pathological data. The three variables of tumor size (log transformed to adjust for non-linearity), log odds of nodal involvement, and stage were retained, but not the number of positive nodes and the number of nodes removed. The SEER area, marital status, and radiotherapy were not significant. The histological groups were jointly significant (P < 0.0001). Their ranking was comparable to Table 3 aside the non-significant SCC microinvasive and carcinoma NOS. We noted that the very large hazard ratio of small cell carcinoma might have inflated the joint significance of histology, hence the model was recomputed excluding it. The histological groups remained jointly significant (P = 0.02).

**Table 4:** Multivariate analysis of cause-specific mortality based on 2,458 cases with complete pathological data

	**A**		**B**	
	**Model including tumor size and nodal variables**		**Model ignoring tumor size and nodal variables**	
**Variable**	**Hazard ratio^(6) ^(95% confidence interval)**	**P**	**Hazard ratio^(6) ^(95% confidence interval)**	**P**

**Pathology**				
Histology ^(1)^		<0.001		<0.001
SCC microinvasive	1.26 (0.40–4.04)	0.693	0.61 (0.19–1.93)	0.407
Carcinoma NOS	0.93 (0.40–2.17)	0.871	1.35 (0.60–3.06)	0.472
Adenocarcinoma excl. mucinous	1.43 (1.07–1.90)	0.016	1.29 (0.97–1.72)	0.078
Adenosquamous carcinoma	1.60 (1.14–2.24)	0.007	1.58 (1.13–2.22)	0.008
Mucinous	1.94 (1.07–3.49)	0.028	2.08 (1.16–3.75)	0.015
Small cell	7.03 (3.95–12.5)	<0.001	7.59 (4.28–13.4)	<0.001
Histological grade 3–4 ^(2)^	1.56 (1.26–1.93)	<0.001	1.83 (1.48–2.26)	<0.001
Log tumor size (mm) ^(3)^	1.89 (1.55–2.31)	<0.001		
Log odds of nodal involvement ^(4)^	1.26 (1.15–1.38)	<0.001		

**Stage**				
Historical stage localized ^(2)^			0.24 (0.19–0.31)	<0.001
Stage II ^(5)^	0.99 (0.62–1.58)	0.966		
Stage III ^(5)^	2.31 (1.70–3.14)	<0.001		
Stage IV ^(5)^	2.57 (1.64–4.04)	<0.001		

**Other**				
Year of diagnosis	0.97 (0.94–0.99)	0.016	0.96 (0.94–0.99)	0.011
African-American ethnicity ^(2)^	1.47 (1.09–1.97)	0.011	1.55 (1.16–2.09)	0.004
Hysterectomy ^(2)^	0.61 (0.47–0.80)	<0.001	0.41 (0.32–0.52)	<0.001

^(1) ^Reference level = non-microinvasive squamous cell carcinoma (SCC), all types.

^(2) ^Binarized variable, coded 1 versus all other levels of the variable including missing.

^(3) ^Untransformed tumor size displayed marked non-linearity.

^(4) ^Log ((number of positive nodes + 0.5)/(number of negative nodes + 0.5)) [14].

^(5) ^AJCC 3rd edition. Reference level = stage I.

^(6) ^Hazard ratio >1 indicates increased risk of dying from cervical cancer.

The analysis was repeated on the same subset in a model that deliberately ignored tumor size and nodal involvement, and used historical stage instead of AJCC stage (Table 4-B). SCC microinvasive and carcinoma NOS remained non significant and the hazard ratios of the other histological types were almost unchanged. Table 4-B suggests that historical stage could be an acceptable surrogate to AJCC staging, but with a drawback: non-mucinous adenocarcinoma was non-significant in the incomplete model (P = 0.078, Table 4-B), whereas it was significant in the complete model (P = 0.016, Table 4-A).

## Discussion

The incidence and mortality rates vary between countries with the highest rates observed in Latin America, South East Asia and Africa. The rates have declined in the last 40 years in industrialized countries, which reflect the success of screening [16-19]. Despite this success, the risks might remain high in the first generation of migrant populations [20-22]. In the United States, the age-standardized incidence and death rates are higher among African-American (11.1 new cases and 5.3 deaths per 100,000) and Hispanic-Latino (15.8 new cases and 3.5 deaths per 100,000) than among White women (8.7 new cases and 2.5 per 100,000) [23]. The incidence of cervical cancer begins to rise at age 20–29 years and increases rapidly to reach a peak usually around age 45–49 years in European populations [1]. The risk of developing an invasive cancer of the cervix is also higher in the age group 40 to 59 years in the US population [23]. The proportion of advanced stage in new invasive cervical cancer was 30% among women aged <50 years and 52% among women aged >= 50 years [24]. For these reasons (worldwide population mobility, risk persistence in migrant populations, higher risk in minorities, early age occurrence, high proportion of advanced stages in newly diagnosed cases), cancer of the cervix is a major healthcare issue even in countries where the incidence has declined.

The present data confirms the overall declining incidence of cervical cancer, but contrasting with an increasing number of adenocarcinomas and adenosquamous carcinomas (Figure 1). Improved classification might have contributed to an apparent increase of adenocarcinomas. Carcinomas that were previously unspecified might have been better characterized in the more recent years, and therefore the increase of adenocarcinomas would reflect only a change in the classifications of the pathological diagnoses. However, the average drop of unspecified carcinomas over three decades was 65/year (= 95–30), whereas the average increase of adenocarcimas was 135/year (non-mucinous 175-80 = 95/year, adenosquamous 45-20 = 25/year, mucinous 15-0 = 15/year), that is, the increase of adenocarcinomas was twice the decline of unspecified carcinomas. Hence a change of classification is insufficient to explain the increase [25]. Many authors have also reported an increasing incidence of adenocarcinomas particularly in younger women [1,2,25-31].

In order to gain an understanding of these trends, we need to consider the current knowledge about the cause(s) of cervical cancer. There is ample experimental and epidemiological evidence that human papillomavirus (HPV) have a key role in cervical carcinogenesis [32-38]. The association of HPV with adenocarcinomas is similar to that with squamous cell carcinomas [39-46], though differing by the role of cofactors, such as tobacco smoking and parity associated with squamous cell carcinomas but not with adenocarcinomas, or oral contraceptives and obesity associated with adenocarcinomas but less so with squamous cell carcinomas [47-52]. If indeed HPV infection is determinant for adenocarcinomas, we expected that the increasing incidence of adenocarcinomas would be preceded by an increasing prevalence of HPV in the general population. We found no nationwide monitoring of HPV prevalence. Nevertheless a most recent survey in 2003–2004 reported that 26.8% of women overall tested positive for one or more strains of HPV [53]. That is a considerable increase as compared to a survey prevalence of 15.1% in 1997–1998 in Arizona among women aged 18–35 years [54], or compared to estimates of 10% global prevalence in the early 1990's [55].

**Figure 4:**
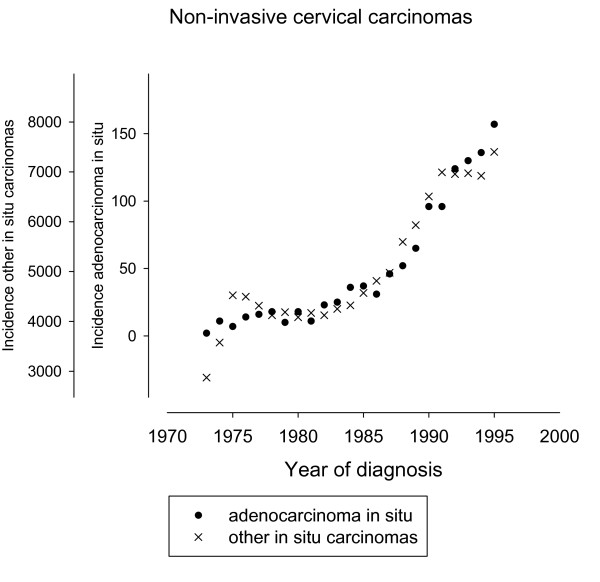
**Numbers of newly diagnosed in situ carcinomas per year**. The plots were centered around the respective means and were scaled by dividing with the respective standard deviations.

We also considered the trends of precursor lesions of adenocarcinomas. The SEER discontinued reporting of cervical in situ carcinomas in 1996, but cases registered between 1973 and 1995 were still available. There were 1,161 in situ adenocarcinoma, versus 116,666 other in situ (51,251 squamous, 47,650 in situ NOS, 17,492 cervical intraepithelial neoplasia grade 3, and 273 miscellaneous). The mean age of the in situ adenocarcinoma patients was 38.2 years, and the mean age of the other in situ patients was 33.3 years. We plotted the respective incidences in Figure 4. The plots were scaled using conventional standardization by subtracting the respective means and dividing by the respective standard deviations. Figure 4 show that in situ adenocarcinoma increased from 10/year around 1975 to 129/year around 1993. The increase was proportional to that of other in situ carcinomas. This similarity of the trends for the in situ carcinomas corroborates the concept of a common etiology. But the very small number of in situ adenocarcinomas as compared to other in situ carcinomas (Figure 4) or compared to invasive adenocarcinomas (Figure 1) suggests that the screening for early glandular lesions had a low sensitivity, and that adenocarcinomas might have been missed by conventional screening, possibly due to their location higher in the cervical canal [31,36,56].

In view of the increasing trend of adenocarcinomas, their association with HPV, their poorer prognosis, and the young age at diagnosis discussed farther below, we argue for the need of improving screening for these tumors and for the opportunity of HPV vaccination [57-61].

Some of the most extensive studies from the literature found that histopathological types were of limited prognostic value, or were significant only in some selected comparisons, or only within some subsets [6,10,62]. In a large study of 11,157 patients from the Patient Care and Evaluation Study comparing adenocarcinoma, squamous cell carcinoma, and adenosquamous carcinoma, Shingleton et al. found no significant differences in 5-year survival among the three tissue types [6]. In an earlier study of the SEER data, no evidence was found of survival differences between squamous cell carcinoma and adenocarcinoma [62]. In an investigation of 17 histological subtypes of non-squamous cell carcinoma of the uterine cervix identified from the Cancer Registry of Norway and histologically verified, Alfsen et al found that histological subtyping lacked significance except small cell carcinoma which was the only histologic subgroup of independent importance for prognosis [10].

These negative results could be interpreted as demonstrating that histopathology genuinely lack in sensitivity for predicting tumor behavior, or caused by wide variation in histopathologic definitions and criteria, lack of standardization in criteria and lack of reproducibility in their application [5]. The lack of reproducibility not only between different observers but also by the same observer was spotlighted in a review of tumor slides from non-squamous cell carcinomas of the uterine cervix [63]. Slides previously reviewed [10] were blindly re-diagnosed more than one year later by the same pathologist (evaluation of intraobserver agreement) and independently by another pathologist (evaluation of interobserver agreement). The overall intraobserver and interobserver kappa index of agreement were respectively 0.53 and 0.44, and the agreement was less than moderate (kappa < 0.40) in respectively 6 and 7 out of 17 histologic types [63]. The agreement was fair to poor for mixed carcinomas and adenocarcinomas NOS, poor for villoglandular and adenosquamous carcinomas, and the distinction of adenocarcinoma in situ from well-differentiated carcinoma proved difficult. Agreement for small cell and undifferentiated carcinomas was moderate (kappa > 0.50) but the pathologists agreed upon the diagnosis only in 2/3 of these diagnoses.

On consideration that the SEER data come from widely differing practices and cover a large time period, there is a priori no reason to believe that the lack of agreement would be any better in the SEER data. Notwithstanding, our analyses find that histology is an important independent prognostic factor. Moreover, the synoptic view of Figure 3 corresponds surprisingly well with what might be expected of prognosis by the ordering of the hazard ratios: the lowest risk microinvasive SCC [64], the good prognosis of endometrioid carcinoma [65], the low risk but uncertain behavior of papillary adenocarcinoma [66], the closely clustered SCC types, the slightly poorer survival of clear cell adenocarcinoma [67], the adenosquamous usually recognized as an aggressive neoplasm [4,68,69], the poor prognosis of mucinous adenocarcinomas [70], and the consistently poorest survival of small cell carcinoma [10]. The histopathological types appear to represent a continuum with largely overlapping confidence intervals which nevertheless do not preclude recognizing distinct clusters (Figure 3). Furthermore the different time trends summarized in Figure 1 suggest that the different histopathologies correspond to distinct biological entities.

Alfsen et al argued that the lack of significance for histopathologic subtyping of adenocarcinomas indicates the need for a simplified nomenclature for these tumors of the uterine cervix [10]. Most likely uncertainty or lack of reproducibility of histopathological subtyping also happened in the SEER data in view of the large confidence intervals (Table 3, 4). This did not preclude the extraction of useful data. Our results argue that histopathologic types identify different tumors, provide valuable prognostic information, and are valuable for the early detection of unexpected trends.

We acknowledge several limitations to this study. Details such as whether the tumors were diagnosed by screening or by symptom occurrence, the type of screening, the HPV status, the status of surgical margins in operated cases, the radiation treatment procedures, the combination or not with chemotherapy, the presence or not of patient's co-morbidities, were not available. The study could not assess to which extent histopathology should affect treatment decision. Factors other than histological type, such as tumor grade, stage or patient's ethnicity, were also important. These factors are not discussed, but clearly they show that the management of the patients cannot be based on histopathology alone. There were many missing data. Only 8% of the cases (2,458 out of 30,989) had full pathological measurements. Even though the concordance of the analyses based on the reduced subsets suggest that the results are robust, the possibility of unknown confounding cannot be excluded. For all these reasons, caution is required, the results should only be considered as explorative.

## Conclusion

In the present study, histological type was found to be an important independent prognostic factor in cervical cancer. The histological types associated with the poorest survival were small cell carcinoma, several subtypes of adenocarcinoma-mucinous, clear cell, and common type of adenocarcinoma- and adenosquamous carcinoma. Incidentally the data confirmed an increasing trend of adenocarcinomas despite an overall decline in the incidence of cervical cancers. The majority of the tumors were diagnosed in women younger than 50 years. The combination of young age, increasing trend, and poorer prognosis of adenocarcinomas questions the efficiency of conventional screening for these tumors, indicate the need to identify women who are at risk and who might most benefit from vaccination.

## Abbreviations

AIC- Akaike Information Criterion. 

AJCC- American Joint Committee on Cancer. 

HPV- human papillomavirus. 

HR- hazard ratio. 

ICDO- International Classification of Diseases for Oncology. 

NOS- not otherwise specified. 

SCC- squamous cell carcinoma. 

SEER- Surveillance, Epidemiology, and End Results.

## Competing interests

The author(s) declare that they have no competing interests.

## Authors' contributions

ATV, VVH: original concept. ATV, CB, GC, MDR, GS, GV: critical appraisal and writing. VVH: responsible for the data integrity and compliance to the SEER Agreement; statistical analyses. All authors read and approved the final manuscript.

## Pre-publication history

The pre-publication history for this paper can be accessed here:



## Supplementary Material

Additional File 1 Check of proportional hazards for the manuscript's models of Tables 3, 4. Figures are rho-values, correlation between residuals and survival time. Rho-values close to 0 indicate less departure from proportional hazards, whereas 1 is the theoretical maximum departure. Click here for file

Additional File 2 Functional forms of the effect of continuous variables on cause-specific mortality. The functional forms were computed from the manuscript's models of Table 3 (left graphs) and Table 4-A (right graphs). Dotted lines: 95% pointwise confidence interval. Click here for file

Additional File 3 Multivariate analysis of 30,989 cases of invasive cancer of the cervix: overall and cause-specific mortality. Results according to the overall and cause-specific mortality endpoints. The hazard ratios are globally comparable; only the cause-specific sub-table is reported in the manuscript. Click here for file
