# Supplementary material for: Prognostic value of histopathology and trends in cervical cancer: a SEER population study
Source: BMC Cancer. 2007 Aug 23;7:164. doi: 10.1186/1471-2407-7-164 (PMC1994954; doi:10.1186/1471-2407-7-164)
Supplement: Additional File 1 — Check of proportional hazards for the manuscript's models of Tables 3, 4. Figures are rho-values, correlation between residuals and survival time. Rho-values close to 0 indicate less departure from proportional hazards, whereas 1 is the theoretical maximum departure. [file 1471-2407-7-164-S1.pdf]

## Additional file 1 - Check of proportional hazards for the manuscript's models of Tables 3-4

Figures are rho-values, correlation between residuals and survival time. Rho-values close to 0 indicate less departure from proportional hazards, whereas 1 is the theoretical maximum departure.

| Variable | Model 3 | Model 4-A | Model 4-B |
|---|---|---|---|
| **Demographics** | | | |
| SEER area | | | |
| Central registries | -0.009 | – | – |
| Western registries | -0.013 | – | – |
| Age at diagnosis | -0.093 ** | – | – |
| Year of diagnosis | 0.014 | 0.004 | -0.009 |
| African-American | 0.009 | 0.103 * | 0.099 |
| Marital status (married) | 0.059 ** | – | – |
| **Pathology** | | | |
| Histology | | | |
| SCC microinvasive | 0.018 | 0.053 | 0.072 |
| Carcinoma NOS | -0.066 ** | 0.096 | 0.062 |
| Adeno. excl. muc. | 0.018 | 0.148 ** | 0.144 ** |
| Adenosquamous | -0.007 | 0.045 | 0.060 |
| Mucinous | 0.026 * | 0.090 | 0.064 |
| Small cell | -0.021 | -0.006 | -0.013 |
| Hiigh grade | 0.009 | -0.089 | -0.105 * |
| Log tumor size | – | -0.118 * | – |
| Log odds nodal involvement | – | 0.026 | – |
| **Stage** | | | |
| Localized | 0.119 ** | – | -0.098 |
| Stage II | – | -0.006 | – |
| Stage III | – | 0.100 * | – |
| Stage IV | – | 0.023 | – |
| **Treatments-interactions** | | | |
| Hysterectomy (HRT) | 0.121 ** | 0.121 * | 0.212 ** |
| Radiotherapy (RT) | 0.178 ** | – | – |
| HRT * RT | -0.076 ** | – | – |
| High grade * RT | -0.026 * | – | – |
| Localized * HRT | -0.056 ** | – | – |
| Localized * RT | -0.069 ** | – | – |
| Localized * HRT * RT | 0.031 ** | – | – |

* $P \leq 0.05$; ** $P \leq 0.01$; – variable not in model.
