# Supplementary material for: Prognostic value of histopathology and trends in cervical cancer: a SEER population study
Source: BMC Cancer. 2007 Aug 23;7:164. doi: 10.1186/1471-2407-7-164 (PMC1994954; doi:10.1186/1471-2407-7-164)

Additional file 2 - Functional forms of the effect of continuous variables on cause-specific mortality

The functional forms were computed from the manuscript's models of Table 3 (left graphs) and Table 4-A (right graphs). Dotted lines: 95% pointwise confidence interval.

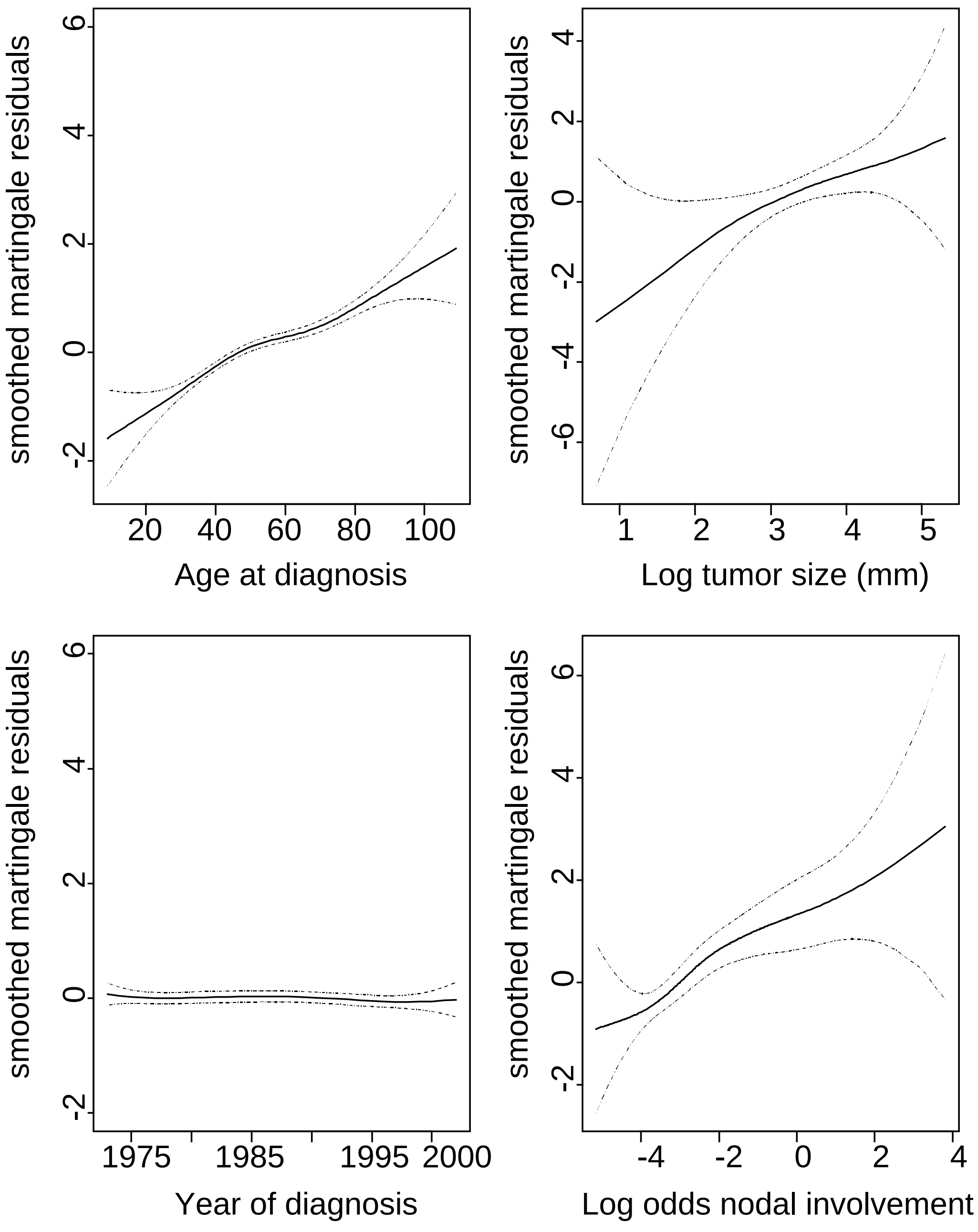

Supplement: Additional File 2 — Functional forms of the effect of continuous variables on cause-specific mortality. The functional forms were computed from the manuscript's models of Table 3 (left graphs) and Table 4-A (right graphs). Dotted lines: 95% pointwise confidence interval. [file 1471-2407-7-164-S2.pdf]
