# Supplementary material for: Prognostic value of histopathology and trends in cervical cancer: a SEER population study
Source: BMC Cancer. 2007 Aug 23;7:164. doi: 10.1186/1471-2407-7-164 (PMC1994954; doi:10.1186/1471-2407-7-164)
Supplement: Additional File 3 — Multivariate analysis of 30,989 cases of invasive cancer of the cervix: overall and cause-specific mortality. Results according to the overall and cause-specific mortality endpoints. The hazard ratios are globally comparable; only the cause-specific sub-table is reported in the manuscript. [file 1471-2407-7-164-S3.pdf]

Additional file 3 - Multivariate analysis of 30,989 cases of invasive cancer of the cervix: overall and cause-specific mortality

Results according to the overall and cause-specific mortality endpoints. The hazard ratios are globally comparable; only the cause-specific sub-table is reported in the manuscript.

	A		B	
	Overall Mortality		Cause-Specific Mortality	
Variable	Hazard ratio[3] (95% confidence interval)	P	Hazard ratio[3] (95% confidence interval)	P
**Demographic characteristics**				
SEER area				
Central registries	0.93 (0.88-0.96)	0.001	0.95 (0.89-1.01)	0.099
Western registries	0.92 (0.88-0.95)	<0.001	0.97 (0.91-1.03)	0.308
Age at diagnosis	1.03 (1.03-1.03)	<0.001	1.01 (1.00-1.01)	<0.001
Year of diagnosis	1.01 (1.00-1.00)	<0.001	1.00 (1.00-1.01)	0.013
African-American ethnicity [1]	1.18 (1.12-1.23)	<0.001	1.14 (1.07-1.22)	<0.001
Marital status (married) [1]	0.93 (0.89-0.96)	<0.001	0.99 (0.94-1.04)	0.638
**Pathology**				
Histological type [2]		<0.001		<0.001
SCC microinvasive	0.84 (0.75-0.93)	0.001	0.28 (0.20-0.39)	<0.001
Carcinoma not otherwise specified	0.95 (0.87-1.03)	0.219	0.91 (0.79-1.04)	0.168
Adenocarcinoma excl. mucinous	1.08 (1.02-1.14)	0.006	1.06 (0.98-1.15)	0.126
Adenosquamous carcinoma	1.28 (1.17-1.40)	<0.001	1.35 (1.20-1.51)	<0.001
Mucinous	1.37 (1.16-1.59)	<0.001	1.52 (1.23-1.88)	<0.001
Small cell	1.66 (1.39-1.96)	<0.001	1.94 (1.58-2.39)	<0.001
Histological High grade [1]	1.61 (1.48-1.74)	<0.001	2.17 (1.93-2.44)	<0.001
Localized stage [1]	0.22 (0.20-0.23)	<0.001	0.07 (0.05-0.08)	<0.001
**Treatments and interactions**				
Hysterectomy (HRT) [1]	0.34 (0.29-0.38)	<0.001	0.26 (0.21-0.31)	<0.001
Radiotherapy (RT) [1]	0.86 (0.80-0.92)	<0.001	1.01 (0.92-1.11)	0.810
HRT * RT	1.86 (1.59-2.17)	<0.001	2.05 (1.67-2.52)	<0.001
High grade * RT	0.75 (0.68-0.81)	<0.001	0.60 (0.53-0.69)	<0.001
Localized stage * HRT	1.51 (1.27-1.77)	<0.001	2.87 (2.17-3.78)	<0.001
Localized stage * RT	2.50 (2.27-2.73)	<0.001	5.24 (4.36-6.29)	<0.001
Localized stage * HRT * RT	0.58 (0.46-0.72)	<0.001	0.40 (0.28-0.56)	<0.001

[1] Binarized variable, coded 1, versus all other levels of the variable including missing.
[2] Reference level = non-microinvasive squamous cell carcinoma (SCC).
[3] Hazard ratio >1 indicates increased risk of death.
